# Direct and Indirect Selection for Grain Yield and Grain Weight in Late Generations of Bread Wheat under Drought Stress and Normal Irrigation Environments

**DOI:** 10.3390/plants11121604

**Published:** 2022-06-18

**Authors:** Rasha E. Mahdy, Dikhnah Ashehri, Hanan Ali Alatawi, Hadba Al-Amrah, Ezzat E. Mahdy

**Affiliations:** 1 Department of Agronomy-Plant Breeding, Faculty of Agriculture, Assiut University, Assiut 71515, Egypt; emahdy@aun.edu.eg; 2 Department of Biology, Faculty of Science, Tabuk University, Tabuk 71491, Saudi Arabia; dalshehri@ut.edu.sa; 3 Department of Biological Sciences, University College of Haqel, Tabuk University, Tabuk 71491, Saudi Arabia; halatwi@ut.edu.sa; 4 Department of Biological Sciences, Faculty of Science, King Abdulaziz University, Jeddah 21589, Saudi Arabia; hggaber@kau.edu.sa

**Keywords:** pedigree selection, *Triticum aestivum*, drought stress, heritability, genotypic correlation, observed gain

## Abstract

Two cycles of pedigree selection for grain yield/plant (GY/P) and grain weight (GW) (100-grain weight) were imposed under drought stress and normal irrigation to study the direct and indirect selection of GY/P and GW in bread wheat. The selection started in the F_6_-generation (Cycle_0_-C_0_) of bread wheat (*Triticum aestivum* L.) traced back to the cross (*Giza 164/Sids 4*) of two Egyptian cultivars. The narrow sense heritability was higher under drought than under normal irrigation and increased by selection. Under drought, the observed direct gain after two cycles of selection for GW was 21.51% (*p* ≤ 0.01), and accompanied with an indirect gain in GY/P of 15.52%. The observed direct gain for GY/P was 17.98% and the indirect gain in GW was 13.81%. Under normal irrigation, the observed direct gain for GW was 12.86% and the indirect gain for GY/P was 16.04%. The direct gain in GY/P was 16.04% and the indirect gain in GW was 11.95%. The genotypic correlations were different in both environments before and after selection. Single trait selection was effective in improving the selection criterion, and selection greatly affected gene associations.

## 1. Introduction

Wheat is a crop of global significance grown in diverse environments. It plays an important role in global food and nutritional security. The cultivation of wheat began 11,000–10,000 years ago [1,2]. Drought is a serious abiotic stress limitation of wheat production in the Mediterranean region and all over the world [3]. It affects physiological and biochemical processes in plants and limits crop production [4]. The Renaissances Ethiopian dam and other dams in the Sudan and Uganda will affect Egypt in terms of water scarcity for agriculture. Therefore, developing high yielding cultivars tolerant to drought stress is one of the main goals of most wheat breeding programs [5]. Pedigree selection for grain yield (GY) has been was practiced by many breeders [6,7,8,9,10,11,12,13,14]. Selection for GY was preferred in late rather than early segregating generations [15] after the plants reached an acceptable level of homozygosity, either under drought or normal irrigation. Two cycles of selection for GY, starting from F_3_ [16], achieved genetic gains in GY of 14.64, 14.91, and 12.12% from the bulk sample in favorable, stress, and other environments, respectively. However, selection for yield was accompanied with lateness in days to heading and a decrease in seed weight.

The low genetic variation under water stress complicates selection for tolerant lines. Refs. [17,18] noted a greater reduction in heritability of most complex traits under drought than under irrigation conditions, which may be due to the decrease in genetic variation and the influence of the genotype by drought interaction. Ref. [19] noted a gain after one cycle of selection in the spike length of 13.43% under favorable conditions, and 8.66% in the bulk sample under heat stress conditions. The realized heritability of the spike length was higher under favorable conditions (0.25–0.56) than under stress (0.18–0.41). The correlated response in GY/spike was 25.35% under favorable conditions and 13.65% in heat stress environments. The observed direct gain in the number of grains/spikes was 14.65% under heat stress when selection was practiced on the F_3_-generation. Several studies revealed positive effects of larger seed size on wheat germination and establishment [20,21,22]. The use of high-quality seed and a large seed size influences seed germination, emergence, seedling growth, establishment, and grain yield [23]. Two cycles of selection for high 1000-grain weight resulted in higher GY than direct selection for GY [13], and the genotypic correlation of GY with the weight of spikes/plant was 0.81, that for the number of grains/spikes was 0.71, that for the weight of grains/spike was “0.91”, that for 1000-grain weight was 0.92, and that for spikelet fertility was 0.70.

Many authors have studied the correlations among yield and its components. The high correlations among yield and yield component traits suggest that selection for yield components can effectively improve yield [24,25]. Grain yield was found to have significant positive correlations with days to maturity, plant height, kernels spike^−1^, and thousand-grain weight, at both genotypic and phenotypic levels [26,27,28,29]. The aim of this study was to examine and compare the observed direct and indirect gains from selection in grain weight and grain yield, and the effect of selection on genotypic correlations among traits in late generations.

## 2. Results

### 2.1. Means and Variability in the F_6_-Generation (Base Population)

The analysis of variance in the F_6_-generation indicated significant differences (*p* ≤ 0.01) among the families, either under drought stress or normal irrigation (Table 1). The genotypic (GCV) and phenotypic coefficients of variation (PCV) were very close in both environments because of the small error variance. The GCV% can be considered medium for days to heading (DH) and plant height (PH), whereas it was high for the other traits.

### 2.2. Effect of Selection on Variability and Heritability of the Selection Criteria

The genotypic and phenotypic coefficients of variation (Table 2) of the selection criteria, i.e., GW (100-grain weight) and GY/P (grain yield/plant) were slightly higher under drought stress than at normal irrigation in F_6_-, F_7_- (C_1_), and F_8_-generations (C_2_), and decreased in successive cycles. Under drought, the GCV for GW decreased from 16.64% in F_6_ to 8.58% in the F_8_-generation (C_2_), and decreased for GY/P from 31.81% to 18.86% in the respective generations.

The narrow sense heritability was higher under drought than under normal irrigation and increased for GW from 71.24 and 53.52% in F_7_ to 87.84 and 76.29% in the F_8_-generation under drought and irrigation, respectively, and increased for GY/P from 29.60 and 28.92% in F_7_ to 76.29 and 72.19% in the F_8_-generation under the respective environments. A wide difference between broad and narrow sense heritability was observed for the two selection criteria under both environments, indicating the presence of dominance and/or epistatic effects.

### 2.3. Mean, Observed, and Correlated Genetic Gains in the Two Cycles of Selection

The performance of the selected families for different traits (Table 3) in the F_7_- and F_8_-generations was lower under drought stress than under normal irrigation. Under drought stress, a significant (*p* ≤ 0.01) direct gain from the mid-parent in the GW increased from 16.96% in C1 to 21.51% in C2. Significant (*p* ≤ 0.05 *p* ≤ 0.01) correlated gains from the mid-parent in the other traits after the second cycle were 5.56% for SL, 12.05% for NS/P, 15.52% for GY/P,15.94% for NG/S and 18.06% for MSW. The observed direct gain from the better parent after two cycles of selection for GW was 13.01% (*p* ≤ 0.01), accompanied with an insignificant indirect gain in GY/P of 8.84%.

The direct gain in GY/P from the mid-parent under drought stress increased significantly (*p* ≤ 0.01) from 15.34% in C1 to 17.98% in C2. Two cycles of selection for GY/P were accompanied with an unfavorable significantly correlated gain in MP (5.25%) and SL (−6.67%), whereas favorable correlated gains from the mid-parent were observed in PH (9.91%), NS/P (15.30%), NG/S (13.36%), MSW (12.40%), and GW (13.81%). Two cycles of selection for GY/P increased GY/P by 11.16% and GW by 5.85% of the better parent (*p* ≤ 0.01), followed by a decrease in SL (−20.0) and an unfavorable increase in DH (15.78%)

It should be mentioned that two cycles of selection under drought stress significantly increased the selection criterion and correlated traits; otherwise, some traits declined.

Under normal irrigation, selection for the GW significantly (*p* ≤ 0.05 *p* ≤ 0.01) increased GW, MSW, GY/P, and NS/P from the mid-parent from C1 to C2. However, after C2, the observed gain from the better parent in GW was not significant (6.41%), and was significant for MSW (7.76%) and NG/S (11.40%), followed by significant adverse effects on NS/P (−9.91%), SL (−9.89%), and DH (4.54%).

Selection for GY/P under irrigation significantly increased GY/P, PH, NS/P, NG/S, MSW, and GW from C1 to C2 in terms of the percentage of the mid-parent. Furthermore, after C2, significant favorable observed gains from the better parent were found in PH (13.33%), NG/S (11.51%), and GW (5.84%). Adverse significant correlated gains from the better parent were observed for DH (6.25%), SL (−18.43%), and NS/P (−8.19%). The direct gain in GY/P was negligible (−0.54%).

### 2.4. Effect of Selection on Genotypic Correlation

It should be noted that the population under study stemmed from the cross Giza164/Sids4. Giza164 is taller, has greater tillering ability and grain yield, and lower GW, MSW, and NG/S, and matures later than Sids4 (Table 1). In the F_6_-generation, under drought stress, DH showed negative genotypic correlations with both SL (−0.4908) and GW (−0.2408), and positive correlations with NS/P and GY/P (Table 4). Furthermore, GY/P yielded positive correlations with NS/P, NG/S, and MSW, a negative correlation with SL, and a weak correlation with GW. This indicates that the late mature families had high yields, NS/P, and NG/S, and selection for GY/P may be more effective to maximize these traits. By comparison, under normal irrigation, DH had negative correlations with GY/P, NG/S, MSW, and GW, indicating that the late mature families were low in yield, NG/S, and MSW. However, GY/P showed positive correlations with PH, NS/P, NG/P, and MSW. Therefore, in both environments, with the exception of SL, selection for GY/P may improve yield components, i.e., NS/P, NG/P, and MSW. It can be concluded that the gene associations were different under both environments. However, selection may alter gene associations.

After two cycles of selection for GW under drought stress, the genotypic correlation of GW with the other traits was weakened, with the exception of that with MSW, which increased from 0.0785 in F_6_ to 0.2341 in the F_8_-generation, and that with NG/S, which changed from −0.6142 to 0.1616 after selection (Table 5). Furthermore, the correlation of GW with the other traits decreased, with the exception of that with MSW. The decrease in genotypic correlations of GW with DH and SL indicated that selection favored the recombinants between the two parents.

Under normal irrigation and selection for GW, the correlations between GW and the other traits were greatly altered in the F_8_-generation. The correlation altered from −0.1316 to −0.7336 for DH, 0.0842 to −0.6864 for PH, 0.1202 to 0.6997 for SL, −0.1627 to −0.3318 for NS/P, 0.0704 to 0.4976 for GY/P, −0.4451 to 0.3867 for NG/S, and 0.1367 to 0.4363 for MSW in the F_6_- and F_8_-generations, respectively. Furthermore, the correlations of GY/P with DH, PH, and NS/P decreased, and that with SL increased. These findings indicate that selection for GW favored the early high yielding (recombinants) families of short PH and high GW. It can be concluded that selection for GW altered the gene associations under both drought stress and normal irrigation.

After two cycles of selection for GY/P (Table 6) in both environments, the genotypic correlations of GY/P with most traits increased or were altered from negative to positive. It can be concluded that the gene associations were different in both environments before and after selection for GW and GY/P, and selection greatly affected gene associations.

## 3. Discussion

In the F_6_-generation (the base population), the GCV% was intermediate for DH and PH, whereas it was high for the other traits, indicating sufficient variability and feasibility of selection (Table 1). The GCV% of GY/P and GW was higher under stress than under irrigation. This may be due to genotype–environment interaction, in which some families can be more greatly affected by drought stress than others, resulting in high variance in these traits. Ref. [30] indicated that the differences between genotypes will inevitably be large under poor or adverse environments, and resistant genotypes can be detected. Refs. [10,15] noted higher genetic variability under drought than under irrigation. In addition, [16,18] reported that the variation decreased under drought stress because of the influence of the genotype by drought interaction.

Selection for GW and GY/P decreased the GCV% from F_6_ to the F_8_-generation in both environments. In late generations, selection from successive cycles for GW and GY/P resulted in homogeneity of the families towards homozygosity, and the differences in the selected families disappeared in successive cycles, consequently reducing PCV and GCV. These results agree with those reported by many authors [8,9,10,12,13,14,15,31,32]. 

A wide difference between broad and narrow sense heritability indicates the presence of dominance and epistatic effects in the F_8_-generation.

The increase in the similarity of the selected families of the traits under selection pressure, and the consequent decrease in the selection differential, resulted in an increase in narrow sense heritability from C1 to C2. Ref. [17] noted heritability for grain yield of 0.20 under irrigation and 0.94 under rain-fed conditions. These results agree with those reported by [9,10,15,16,31]. 

With respect to the observed genetic gain, many authors have suggested indirect selection for grain yield because of grains polygenic nature, low heritability, linkage, and genotype–environment interaction [13,32,33,34,35], and selection for morphological and physiological traits may improve wheat grain yield in diverse environmental conditions [36,37]. By comparison, the present study indicated that the observed gain from direct selection for GY/P under drought stress was 17.98%, and 16.04% for the mid-parent under normal irrigation, in the F_8_-generation. In addition, the indirect gain in GY/P via selection for GW was 15.52% under drought and 11.07% under irrigation. Furthermore, the observed gain was greater under drought stress than under irrigation. This may be due to the high GCV%, PCV%, and heritability under stress than in an optimum environment. These results imply that selection for GY/P and GW under drought stress (antagonistic selection = selection and environment act in opposing directions) was more effective than under normal irrigation (synergistic selection = selection and environment act in the same direction) in improving GY/P and GW. This is consistent with the findings of [38], who demonstrated that antagonistic selection is better than synergistic selection in changing the mean. Other studies have reached the same conclusion [10,15,16].

With respect to genotypic correlations among traits in the F_6_-generation (Table 4), the results indicated that the correlations differed more under drought stress than under irrigation. Selection for GW increased NG/S, GY/P, and MSW, and decreased DH, in both environments, whereas selection for GY/P increased DH, PH, NS/P, NG/S, and MSW. It can be concluded that selection for GW and GY/P altered the gene associations under both drought stress and normal irrigation. Refs. [24,25] indicated that the high correlations among yield and yield component traits suggest that selection for yield components can effectively improve yield. Grain yield had significant positive correlations with days to maturity, plant height, kernels/spike, and thousand-seed weight, at both genotypic and phenotypic levels [26,27,28,29].

It can be concluded that the genotypic coefficient of variability in these materials was higher under drought than under irrigation, and decreased from the F_6_-generation to the F_8_-generation. Narrow sense heritability was higher under drought than under normal irrigation. The performance of the selected families for different traits in the F_7_- and F_8_-generations was lower under drought stress than under normal irrigation. Single trait selection was effective in improving the selection criterion and accompanied with adverse effects on some correlated traits. The genotypic correlations were different in both environments before and after selection, and selection greatly affected gene associations.

## 4. Materials and Methods

Two cycles of pedigree selection were performed. The selection criteria were grain yield/plant (GY/P) and 100-grain weight (GW). The plant genetic materials were families in the F_6_-generation of bread wheat (*Triticum aestivum* L.) traced back to the cross (Giza 164/Sids 4) of two Egyptian cultivars. The pedigree of *Giza 164* is “*KVZ/Buha“S”//K al/Bb*”, and that of *Sids 4* is “*Maya“S” Man (S)//CMH74.AS92/3/Giza157-2*”. These materials comprised 100 families derived from a previous study of selection in early generations (F_2_–F_5_) under drought stress, and another 100 families derived from selection under normal irrigation [15]. Two experiments, one under drought stress and the other under normal irrigation, were conducted in each season. The experiments in the three seasons (2017/2018, 2019/2020, and 2020/2021) were conducted at the Faculty of Agriculture Experimental farm, Assiut University, Egypt (Longitude:31.125° Latitude: 27.25° E, Elevation: 45 m/148 Feet). Planting dates were between 20 November and 25 November in the three seasons. A randomized complete block design with three replications was used. Super phosphate (P_2_O_5_, 15.5%) was added during land preparation at a rate of 357.14 kg/ha. Before the first irrigation, nitrogen fertilization was added in one dose in the form of ammonium nitrate (33.5% N) at a rate of 190.5 kg/ha. The experiments under drought stress received planting irrigation and only one irrigation three weeks later, whereas the experiments under normal irrigation received planting irrigation and four irrigations throughout the growing season.

In the season of 2017/2018, 100 families in the F_6_-generation, along with the parents, were sown under drought stress in rows that were three meters long, 30 cm apart. Another 100 families in the F_6_-generation were sown under normal irrigation. The experimental unit was one row. After full emergence, seedings were adjusted to ten plants per meter. The collected data were days to heading (DH), plant height (PH, cm), spike length (SL, cm), number of spikes/plant (NS/P), grain yield/plant (GY/P, g), number of grains/spike (NG/S), main spike weight (MSW, g), and 100-grain weight (GW, g). The same procedure was followed in the second and third seasons. At the end of the season, 20 plants from each family were harvested. The best 20 families in GY/P and GW were identified in both experiments, and the best plant from each was saved for the next season. In the second season, of 2019/2020, the 20 families selected for GY/P and GW, along with the parents, were sown under drought conditions, and the same procedure as that of the irrigation experiment was followed. At the end of the season, the ten best families for each selection criterion from both experiments were saved. In the third season, 2020/2021, (F_8_-generation) the selected families were evaluated under their respective environments.

### Biometrical Analysis

The analyses of variance, covariance, phenotypic variance (σ^2^p), and genotypic variance (σ^2^g), and significance tests, were performed as in [39] on a plot mean basis. The mathematical model of the randomized complete block design is:

Yij = μ + ηi + ξj + eij

where i = 1,2,3,…, t and j = 1,2,…, b with t treatments and b blocks. μ is the overall mean based on all observations, ηi is the effect of the ith treatment response, ξj is the effect of the jth block, and eij is the corresponding error term, which is assumed to be independent and normally distributed with mean zero and constant variance.

In the random model of the RCBD, genotypic variance (σ^2^g) = (MSg − MSe)/r, and phenotypic variance (σ^2^p) = σ^2^g + MSe/r, where MSg = genotype mean square, MSe = error mean square, r = number of replications.

The phenotypic (PCV) and genotypic (GCV) coefficients of variation and the genotypic correlations among pairs of traits were estimated as outlined by [40] as follows:

The covariance components were used to compute the genotypic correlation on a line mean basis, between the various characters as follows:

rg = σ_g1.2_/√((σ^2^_g1_) (σ^2^_g2_))

where σ_g1.2_ is the genetic covariance between trait 1 and trait 2, and σ^2^g is the genetic variance.

GCV% = (σ_g_/mean) × 100, PCV% = (σ_p_/mean) × 100

where σ_g_ and σ_p_ = genotypic and phenotypic standard deviations, respectively.

Heritability in the broad sense (H) and the genetic advance were computed using the formula adopted by [41] as follows:

Heritability in the broad sense (H%) = (σ^2^_g_/σ^2^_p_) × 100 and the expected genetic gain is:

F_2_ = k* σ_p_* H

where the environmental variance σ^2^_E_ = (σ^2^_P1_ + σ^2^_P2_)/2, σ^2^_p_ = F_2_ variance, σ^2^_g_ = σ^2^_p_ − σ^2^_E_, σ^2^_P1_ = variance of the first parent, σ^2^_P2_ = phenotypic variance of the second parent, k is the selection intensity from selecting 10% of the superior plants.

Heritability in the narrow sense (h^2^) was estimated by parent–offspring regression as outlined by [42].

The observed genetic gain was calculated as a percentage from the mid- and better parent. The significance of the direct and correlated observed genetic gains was calculated using the least significant difference (LSD):

LSD for mid-parent observed gain = t_α_ ((MSE/(r × f) + MSE/(r × 2))^0.5^,

LSD for better parent observed gain = t_α_ ((MSE/(r × f) + MSE/r)^0.5^,

where t_α_ = tabulated t at 0.05 or 0.01 level of probability, r = number of replications, MSE = error mean squares, and f = number of families.

## 5. Conclusions

The GCV% of GY/P and 100-GW was higher under stress than under irrigation. This may be due to genotype–environment interaction, in which some families can be more significantly affected by drought stress than others, thus resulting in high variance. Alanis and Hill (1966) noted that the differences between genotypes will inevitably be large in poor or adverse environments, and resistant genotypes can be detected. The PCV and GCV decreased by selection from successive cycles towards homozygosity because of the increase in the similarity of the selected families of the traits under selection pressure. The narrow sense heritability increased from C_1_ to C_2_ in late generations. Furthermore, the observed gain was better under drought stress than under irrigation. The direct observed genetic gain was better than the correlated gain in both environments. Single trait selection was effective in improving the selection criterion, and was accompanied with adverse effects on some correlated traits in both environments. Furthermore, the observed gain was better under drought stress than under irrigation. The results imply that antagonistic selection was better than synergistic selection in improving GY/P and 100-GW. Selection for 100-GW increased NG/S, GY/P, and MSW, and decreased DH, in both environments, whereas selection for GY/P increased DH, PH, NS/P, NG/S, and MSW. The genotypic correlations among traits differed more under drought than under irrigation.

## Figures and Tables

**Table 1:** Parental mean, mean squares, and genotypic (GCV%) and phenotypic (PCV%) coefficients of variability of traits in the base population (F_6_) under drought stress and normal irrigation.

Drought Stress								
	DH	PH	SL	NS/P	GY/P	NG/S	MSW	GW
Reps	34.93	234.04	31.55	1.71	8.03	585.56	0.68	1.53
Families	87.10 **	199.07 **	12.01 **	9.18 **	22.82 **	378.90 **	0.56 **	1.35 **
Error	1.80	17.01	0.48	0.14	2.31	78.91	0.13	0.01
GCV%	7.00	8.30	14.69	29.87	31.81	25.98	24.90	16.64
PCV%	7.08	8.68	15.00	30.11	33.55	29.20	28.45	16.67
Sids4	68.0	80.0	14.67	5.00	7.00	40.00	2.10	4.05
Giza164	86.0	90.0	10.0	6.33	8.67	35.00	1.52	3.36
Normal Irrigation								
Reps	5.02	664.20	11.78	77.13	143.88	420.83	19.43	8.54
Families	103.43 **	246.14 **	14.23 **	8.20 **	19.09 **	579.75 **	1.42 **	1.43 **
Error	1.69	16.27	0.91	0.19	2.69	1.07	0.02	0.01
GCV%	7.41	9.03	15.31	22.77	21.25	27.55	27.09	14.52
PCV%	7.47	9.35	15.83	23.03	22.93	27.57	27.24	14.56
Sids4	68.0	90.0	16.0	6.33	8.90	56.33	2.30	5.60
Giza164	83.0	100.0	11.33	8.00	11.67	41.33	1.60	4.60

** significant at 0.01 level of probability. DH = days heading, PH = plant height, SL = spike length, NS/P = number of spikes/plant, GY/P = grain yield/plant, NG/S = number of grains/spikes, MSW = main spike weight, GW = 100-grain weight.

**Table 2:** Genotypic (GCV%) and phenotypic (PCV%) coefficients of variation, and heritability in the broad (H%) and narrow sense (h^2^%) for traits under selection pressure.

Selection Criterion	Selection Cycle	Item	Drought Stress	Normal Irrigation
grain weight	C_0_ (F_6_)	GCV%	16.64	14.52
		PCV%	16.67	14.56
		H%	88.57	99.37
		h^2^%	-	-
GY/P	C_0_ (F_6_)	GCV%	31.81	21.25
		PCV%	33.55	22.93
		H%	89.88	85.89
		h^2^%	-	-
100-grain weight	C_1_ C_1_ (F_7_)	GCV%	13.44	12.46
		PCV%	13.47	12.47
		H%	99.58	99.94
		h^2^%	71.24	53.52
	C_2_ C_2_ (F_8_)	GCV%	8.58	8.27
		PCV%	9.05	8.8
		H%	89.97	88.26
		h^2^%	87.84	76.29
GY/P	C_1_ C_1_ (F_7_)	GCV%	29.94	10.05
		PCV%	30.50	11.82
		H%	96.37	72.35
		h^2^%	29.60	28.92
	C_2_ C_2_ (F_8_)	GCV%	18.86	18.61
		PCV%	19.14	19.02
		H%	94.57	98.29
		h^2^%	76.29	72.19

**Table 3:** Mean, direct, and correlated observed genetic gains from selection as a percentage of the mid-parent (MP OG%) and the better parent (BP OG%) for GW and GY/P in cycle 1 (C_1_) and cycle 2 (C_2_) under drought stress and normal irrigation.

Selection Criterion	Selection Cycle	Drought Stress							
		DH	PH	SL	NS/P	GY/P	NG/S	MSW	GW
GW	Mean C1	77.53	71.42	12.60	6.73	7.80	44.00	1.49	4.74
	MP OG%	0.69	−1.49	5.00	9.46 **	14.71 *	14.29 **	14.59 **	16.96 **
	BP OG%	10.76 **	−10.73 **	−10 **	−3.83	0.00	4.76	−0.69	7.66 **
	Mean C2	83.67	87.17	12.67	6.28	8.00	40.00	1.48	5.65
	MP OG%	1.41	−0.38	5.56 **	12.05 **	15.52 *	15.94 **	18.06 **	21.51 **
	BP OG%	11.56 **	−3.15	−9.52	1.21	8.84	2.56	13.41 *	13.01 **
GY/P	Mean C1	80.30	76.17	10.70	7.02	7.84	42.78	1.44	4.57
	MP OG%	4.29 **	5.06	14.00 **	14.09 **	15.24 **	11.11 **	10.98 *	12.01 **
	BP OG%	14.71 **	4.79	4.00 *	0.24	0.47	1.84	−3.82	2.40
	Mean C2	86.83	96.17	11.20	6.42	8.17	39.11	1.41	5.29
	MP OG%	5.25 **	9.91 **	−6.67 *	15.30 **	17.98 **	13.36 **	12.40 *	13.81 **
	BP OG%	15.78 **	6.85	−20 **	4.67	11.16 **	0.28	8.00	5.85 **
Normal irrigation									
GW	Mean C1	80.60	90.73	13.55	9.28	17.73	63.20	3.26	5.75
	MP OG%	3.33 **	0.81	4.19 **	9.20 *	10.82 *	10.32 *	11.99 **	12.79 **
	BP OG%	11.94 **	−4.49	−9.70 **	−19.99 **	−1.44	15.82 *	8.61 **	4.58 **
	Mean C2	83.63	91.50	13.22	6.80	16.09	51.85	2.76	5.85
	MP OG%	0.16	0.73	2.99	11.05 **	11.07 *	12.20 **	15.10 **	12.86 **
	BP OG%	4.54 *	−3.68	−9.89 **	−9.91*	−4.23	11.40 **	7.76 **	6.41
GY/P	Mean C1	84.43	93.90	12.37	9.92	18.24	45.20	2.30	5.66
	MP OG%	8.25 **	4.33 *	−5.14 **	12.06 **	14.00 **	12.14 *	10.83 *	10.91 **
	BP OG%	17.27 **	−1.16	−17.79 **	−17.8 **	1.33	−4.34	−11.39	2.84 **
	Mean C2	85.00	98.22	11.96	6.93	16.71	51.92	2.68	5.82
	MP OG%	1.80	8.13 **	−6.78	13.17 **	16.04 **	12.34 **	12.05 **	11.95 *
	BP OG%	6.25 *	13.33 **	−18.43 **	−8.19 *	−0.54	11.53 **	4.88	5.84 *

*, ** significant at 0.05 and 0.01 level of probability, respectively. DH = days heading, PH = plant height, SL = spike length, NS/P = number of spikes/plant, GY/P = grain yield/plant, NG/S = number of grains/spike, MSW = main spike weight, GW = 100-grain weight.

**Table 4:** Genotypic correlations among traits in the base population (F_6_) under drought stress (above diagonal) and normal irrigation (below diagonal).

Trait	DH	PH	SL	NS/P	GY/P	NG/S	MSW	GW
DH		0.1922	−0.4908	0.3685	0.3126	0.1862	0.0463	−0.4908
PH	0.0971		−0.0880	0.1932	0.0336	−0.1627	−0.1295	0.0507
SL	−0.5025	0.0761		−0.3598	−0.2955	−0.1531	0.0055	0.2762
NS/P	0.3215	0.1660	−0.3649		0.2540	−0.2762	−0.3519	−0.1374
GY/P	−0.1446	0.4930	−0.2551	0.3081		0.3537	0.3707	−0.0447
NG/S	−0.0863	0.0037	0.2972	−0.2729	0.1413		0.8368	−0.6142
MSW	−0.0973	0.0712	0.2021	−0.1672	0.2107	0.0329		0.0785
GW	−0.1316	0.0842	0.1202	−0.1627	0.0704	−0.4451	0.1367	

DH = days heading, PH = plant height, SL = spike length, NS/P = number of spikes/plant, GY/P = grain yield/plant, NG/S = number of grains/spikes, MSW = main spike weight, GW = 100 grain weight.

**Table 5:** Genotypic correlations among traits under drought stress (above diagonal) and under normal irrigation (below diagonal) when selection was practiced for grain weight in the F_8_-generation.

Trait	DH	PH	SL	NS/P	GY/P	NG/S	MSW	GW
DH		0.2659	−0.2337	0.0923	0.2246	0.0071	0.0094	−0.2171
PH	0.9633		−0.3097	0.5834	−0.0047	0.0020	−0.0982	−0.1628
SL	−0.5716	−0.5655		0.9078	−0.0087	0.0036	−0.1815	−0.3009
NS/P	0.8063	0.5747	−0.4302		0.3506	0.0688	0.0886	0.0063
GY/P	−0.7627	−0.7171	0.4654	−0.2913		0.3057	0.6001	−0.0045
NG/S	−0.2249	0.0132	0.4115	−0.5337	0.0894		0.4593	0.1616
MSW	−0.2658	−0.4607	0.3828	−0.4032	0.0080	0.5817		0.2341
GW	−0.7336	−0.6864	0.6997	−0.3318	0.4976	0.3867	0.4363	

DH = days heading, PH = plant height, SL = spike length, NS/P = number of spikes/plant, GY/P = grain yield/plant, NG/S = number of grains/spikes, MSW = main spike weight, GW = 100 grain weight.

**Table 6:** Genotypic correlations among traits under drought stress (above diagonal) and under normal irrigation (below diagonal) when selection was practiced for GY/P in the F_8_-generation.

Trait	DH	PH	SL	NS/P	GY/P	NG/S	MSW	GW
DH		0.3606	−0.2445	0.1601	0.4123	0.1131	0.3710	−0.2778
PH	0.2254		−0.2420	0.2515	0.9558	0.2465	0.5907	−0.4914
SL	−0.1170	−0.3278		−0.4331	0.0121	0.1732	0.1357	0.1325
NS/P	0.2602	0.3795	0.0135		0.4751	−0.2180	−0.1054	0.2107
GY/P	0.8286	0.3617	−0.0367	0.8145		0.3617	0.8152	−0.3061
NG/S	0.8265	−0.0624	0.1903	0.2079	0.5618		0.8501	−0.3826
MSW	0.8392	−0.0403	−0.1259	−0.0202	0.4365	0.2639		−0.4565
GW	0.2431	0.2758	−0.0794	0.4510	0.5994	−0.2782	0.6793	

DH = days heading, PH = plant height, SL = spike length, NS/P = number of spikes/plant, GY/P = grain yield/plant, NG/S = number of grains/spikes, MSW = main spike weight, GW = 100 grain weight.
